# Why are imprints unstable in pluripotent stem cells?

**DOI:** 10.1042/BST20243003

**Published:** 2025-07-17

**Authors:** Maria Arez, Simão Teixeira da Rocha

**Affiliations:** 1 Department of Bioengineering, iBB - Institute for Bioengineering and Biosciences, Instituto Superior Técnico, Universidade de Lisboa, Lisbon, Portugal; 2 Associate Laboratory i4HB Institute for Health and Bioeconomy, Instituto Superior Técnico, Universidade de Lisboa, Lisbon, Portugal

**Keywords:** epigenetics, genomic imprinting, DNA methylation, pluripotency, stem cells

## Abstract

Pluripotent stem cells (PSCs) possess the remarkable ability to self-renew and differentiate into nearly any cell type, making them invaluable for both research and therapeutic applications. Despite these powerful attributes, PSCs are vulnerable to genetic and epigenetic instabilities that can undermine their reliability and safety. While genetic abnormalities can be routinely monitored with established guidelines, epigenetic instabilities often go unchecked. Among the most recurrent epigenetic defects in PSCs are errors in genomic imprinting — a process that governs parent-of-origin-specific monoallelic expression of certain genes through differential marking of the two parental alleles by DNA methylation. When disrupted, it becomes a source of a dozen developmental conditions known as imprinting diseases. In PSCs, once imprinting errors arise, they remain throughout cellular differentiation, casting uncertainty over the use of PSC-derived cells for disease modelling and regenerative medicine. In this review, we provide an overview of imprinting defects in both mouse and human PSCs, delving into their origins and consequences. We also discuss potential correction strategies that aim to enhance imprinting stability, ultimately paving the way for safer, more reliable PSC use in research and clinical applications.

## Introduction

Pluripotent stem cells (PSCs) are notable for their unique abilities to self-renew and differentiate into diverse cell types, making them a promising tool for both research and therapeutics. Indeed, PSC research has achieved several significant milestones, ranging from the isolation of mouse and human embryonic stem cells (hESCs) [1–3] to the generation of induced PSCs (iPSCs) by reprogramming somatic cells to the pluripotent state [4–6]. Recent progress includes the development of blastoids and organoids to study developmental processes and disease mechanisms [7,8] and the numerous clinical trials exploring therapies that utilise PSC-based cell product derivatives [9].

An important bottleneck in stem cell research comes from the genetic and epigenetic instabilities of PSCs, affecting their reliability and safety across their distinct applications [10]. Whereas genetic alterations are well documented and easier to spot [10,11], epigenetic aberrations are often overlooked in routine assessments. These epigenetic changes emerge during derivation and culture of PSCs, while being difficult to track and control. Importantly, these epigenetic defects are often maintained during differentiation of PSCs into specialised cell types [12–14], possibly leading to abnormal cellular behaviour and compromised functionality in PSCs and their derivatives [15–17].

The most well-studied epigenetic anomalies in PSCs include changes in DNA methylation. While many of these changes are reversed upon differentiation, some genomic regions exhibit irreversible alterations. This is particularly true for regions with allelic differences in DNA methylation patterns, such as those influenced by the epigenetic phenomenon of genomic imprinting. These aberrations impact the transcription of several genes having long-lasting impact on pluripotency and general fitness and functionality of PSC-derived differentiated cells [18]. This concise review offers a comprehensive overview of imprinting abnormalities in mouse and human PSCs, examines the underlying factors contributing to these aberrations and discusses potential strategies for their correction.

### Genomic imprinting and its developmental cycle

Genomic imprinting is a mammalian parent-of-origin-specific epigenetic mechanism that controls the monoallelic expression of a subset of genes [19]. Unlike most genes that are expressed from both maternal and paternal alleles, imprinted genes are biased or exclusively expressed from only one parental allele. This results in a functional asymmetry between the maternal and paternal genomes, emphasising the crucial requirement of both parental genomes for proper development [20,21]. Imprinted genes not only regulate growth and development but also metabolism, cognitive and behavioural functions. To date, more than a few hundred imprinted genes have been identified in humans and mice, many of which are imprinted in both species [19].

**Figure 1:**
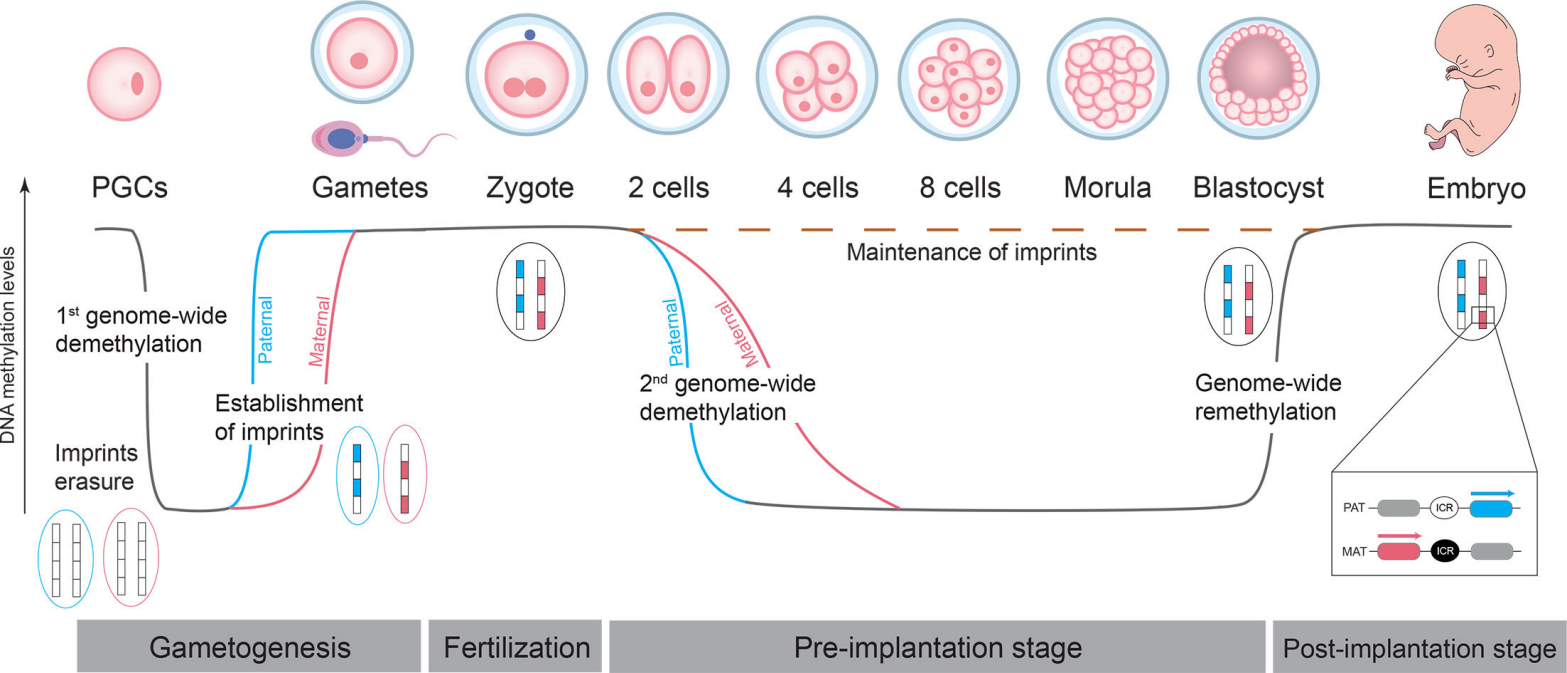
The life cycle of genomic imprinting. During gametogenesis, primordial germ cells (PGCs) undergo genome-wide demethylation, erasing existing imprints. New maternal (red) and paternal imprints (blue) are established in a sex-specific manner in developing gametes and are maintained through fertilization. After fertilization, in the preimplantation stage, a second wave of genome-wide demethylation occurs but imprints are maintained. In the post-implantation stage, the genome undergoes remethylation, while parental-specific imprints remain intact to regulate monoallelic gene expression in the developing embryo. The inset highlights an example of an imprinted cluster with a maternal imprint: whereas the maternal (MAT) imprinted control region (ICR) is methylated (black circle) leading to the silencing of the gene downstream the ICR and the expression of the gene upstream of this region, the paternal (PAT) ICR is unmethylated (white circle) leading to the expression of the gene downstream of the ICR and the silencing of the gene upstream of the ICR.

Parent-of-origin-specific expression of imprinted genes involves known epigenetic modifications such as DNA methylation on their regulatory regions. Most imprinted genes are in close proximity in the genome, organised in clusters that are under control of a *cis*-acting regulatory element called imprinting control region (ICR). ICRs are CpG-rich regions that contain opposing DNA methylation patterns on the maternal and paternal alleles [22–25]. DNA methylation at ICRs, also known as imprints, is set in the germline in a parent-of-origin-dependent manner through the action of the *de novo* DNA methyltransferase DNMT3A in combination with the DNMT3L cofactor [26–28]. During the preimplantation period, cells undergo a genome-wide wave of epigenetic changes such as DNA demethylation (Figure 1). This occurs in two forms: an active one, primarily mediated by the ten-eleven translocation (TET) protein TET3, that demethylates the paternal-inherited DNA material at the first cell stage [29]; and a passive one that demethylates the maternal-inherited DNA material in a replication-dependent manner due to low levels of the maintenance DNA methyltransferase, DNMT1 [30–32] (Figure 1). Remarkably, both maternal and paternal imprints resist this DNA demethylation wave. This resilience occurs via sequence-specific binding factors that recruit epigenetic modifiers to protect imprints from global demethylation. These are the KRAB-zinc finger proteins ZFP57 and ZNF445 that bind to specific sequences in the ICRs in a methylation-dependent manner and interact with the cofactor KAP1 [33,34]. This interaction mediates the recruitment of chromatin modifiers and DNMTs to maintain the methylation marks at the methylated ICR, ensuring the stability of imprinting. Around the time of implantation, a genome-wide wave of re-methylation takes place, during which many genomic regions restore DNA methylation levels, while imprints remain protected, this time, from acquiring *de novo* methylation marks. Imprints are then faithfully propagated to the daughter cells throughout development, across the lifespan and into ageing [35]. It is also around implantation that other regions, besides ICRs, acquire differential DNA methylation. These regions, known as somatic differentially methylated regions (sDMRs), regulate the monoallelic expression of imprinted clusters, but their methylation status [36] depends on the methylation of the ICR of that imprinted cluster. These sDMRs can vary across tissues and developmental stages, driving tissue- and time-specific imprinting patterns [37]. Finally, imprints undergo a complete reset in primordial germ cells during another wave of genome-wide demethylation that is achieved by the combination of a passive demethylation through down-regulation of DNMTs and their cofactors, and an active demethylation mediated by TET1, from which imprints are, this time, unprotected [38–40]. Imprints are then re-established in an oocyte- or sperm-specific manner during germ cell development [26–28], ensuring the cycle continues into subsequent generations (Figure 1).

As noted, genomic imprinting stability relies on a delicate equilibrium of epigenetic modifications that are established, maintained and reprogrammed at various stages of development. Occasionally, the imprinting cycle is disrupted, dysregulating monoallelic expression of imprinted genes, which can result in imprinting disorders affecting growth and development, as well as metabolic and neuronal functions, such as Prader–Willi (PWS), Angelman (AS) and Beckwith–Wiedemann syndromes [41]. Despite occasional failures leading to disease, imprints are robustly stable during mammalian development. The greatest challenge occurs during the preimplantation wave of global DNA demethylation, followed by the establishment of *de novo* patterns of DNA methylation as cells initiate lineage commitment [42]. ESCs, the pluripotent cells of the inner cell mass (ICM) of the blastocyst that will originate all cells in the embryo proper, are at the centre of these dynamic methylation changes. These cells undergo approximately 10 to 15 cell divisions *in vivo* and cope with minimal levels of DNA methylation while maintaining stable imprints. ESCs can be derived and cultured indefinitely *in vitro*, retaining their ability to self-renew and to differentiate into all three embryonic germ layers. The perpetuation of these cells *in vitro* far longer than *in vivo* renders them susceptible to both genetic and epigenetic changes which should not be neglected. The following discussion will examine how DNA methylation and imprints are regulated during *in vitro* culture of ESCs, as well as during the establishment of the PSC state from somatic cells.

### The delicate epigenetic equilibrium of PSCs

PSC lines represent the *in vitro* counterparts of embryonic cells in pre-implantation development. In addition to cells with primordial origin from the pre-implantation embryo, known as ESCs [1–3], PSCs can also originate from somatic cells through the ectopic expression of a cocktail of pluripotent transcription factors, including OCT4, SOX2, KLF4 and c-MYC, that are referred as iPSCs [4–6]. Despite their different origins, ESCs and iPSCs are broadly equivalent in their pluripotent properties and transcriptional profiles [43]. However, iPSCs have been shown to display epigenetic differences compared with ESCs, including the retention of somatic cell memory and reprogramming-associated epigenetic errors, such as disruptions in genomic imprinting [18,44].

**Figure 2:**
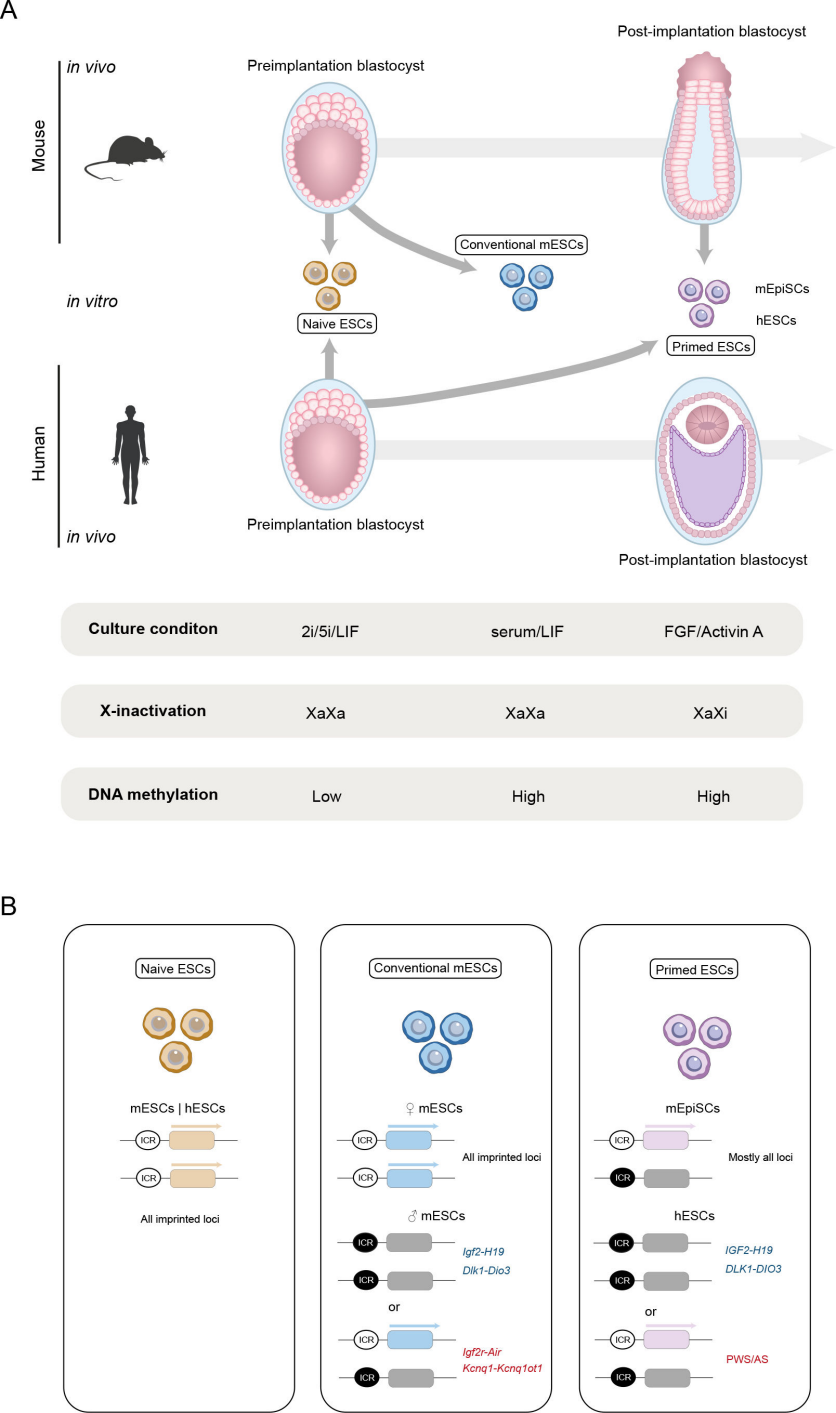
The influence of pluripotency states on genomic imprinting in mouse and human ESCs. **(A**) Schematic representation of the derivation of mouse and human ESCs with different pluripotency states from pre- and post-implantation blastocysts. Key features, including culture conditions, X-chromosome inactivation status (XaXa vs. XaXi), and global DNA methylation levels (low vs. high), are summarized for each state. (**B**) Imprinting status across the different pluripotency states of ESCs. Naive ESCs (both mouse and human) show a complete loss of imprinting methylation at all loci. Conventional mESCs can exhibit different outputs based on the biological sex of the cells. Primed ESCs exhibit greater imprinting stability (mEpiSCs), but in humans, certain imprinted regions tend to be hypermethylated. In blue are represented examples of paternal imprinted loci and in red examples of maternal imprinted loci that typically show the imprinting states/defects depicted (please refer to the main text for other examples); black circles: methylated ICR; white circles: unmethylated ICR. ESCs, embryonic stem cells; ICR, imprinting control region; PWS/AS, Prader–Willi syndrome/ Angelman syndrome imprinted locus.

PSCs can exist in different grades of pluripotency which influence their cellular characteristics, developmental potential and epigenetic landscapes (Figure 2A). The naive state reflects the ground state of pluripotency of the ICM of the pre-implantation embryo. These cells are characterised by a highly undifferentiated state, maximal differentiation potential, low DNA methylation levels and the presence of two active X chromosomes in female cells (XaXa). *In vitro*, the naive state, among subtle formulation variations, is commonly achieved by inhibiting the FGF/ERK and GSK3 signalling pathways in the presence of Leukemia Inhibitory Factor (LIF) [45,46]. On the other side of the pluripotency spectrum is the primed state that corresponds to a later stage of embryonic development, resembling the peri/post-implantation epiblast cells. These cells are characterised by a reduced differentiation potential compared with naive cells, higher DNA methylation levels and the presence of an inactive X chromosome in female cells (XaXi). *In vitro*, this primed state is achieved by supplementation with FGF2 and Activin A, which activate signalling pathways regulating the balance between maintaining pluripotency and being primed to differentiate [3,47,48]. This was the original medium formulation used to derive the first hESCs [3] and remains the conventional medium of choice for human PSC culture [49]. In contrast, for mESCs, conventional culture conditions initially included foetal bovine serum (FBS) with mitotically inactivated mouse embryonic fibroblasts (feeders), followed by a LIF/serum formulation. In this state, mouse PSCs are closer to naive state, with female cells maintaining an XaXa configuration, but they fail to achieve a consistent ground-state pluripotency and exhibit higher DNA methylation levels [49]. Considering the spectrum of pluripotency states and their distinct epigenetic landscapes (Figure 2A), it is not surprising that imprints can be affected and even dysregulated *in vitro*.

Besides the epigenetic differences associated with pluripotency, epigenetic variability can also emerge during somatic cell reprogramming into iPSCs. This process involves rapid and extensive epigenetic rewiring [50,51], and therefore, it is unsurprising that epigenetic anomalies can occur. They originate from distinct sources. First, many reprogramming cells fail to completely erase their somatic identity, resulting in final iPSCs retaining epigenetic memory of their cell of origin. Second, additional epigenetic aberrations can emerge during the reprogramming process itself, further affecting the epigenetic landscape of iPSCs. A notable example is the dysregulation of imprints, which are often affected during reprogramming. In the following sections, we will explore the origins of imprinting defects during the derivation and maintenance of both mouse and human ESCs/iPSCs and discuss possible strategies preventing the emergence of imprinting errors.

### Imprinting status in mouse ESCs

Following the discovery of imprinted genes and imprinting mechanisms, researchers quickly sought to determine whether mouse ESCs (mESCs) could serve as a reliable cellular model for studying imprinting regulation. A word of caution on the stability of imprints soon emerged**,** especially upon prolonged propagation of these cells in culture [52]. For example, in a study by Feil et al. [53], analysing the *Commd1/Zrsr1* locus, the authors observed hypomethylation of the ICR and consequent biallelic expression of *Zrsr1* (formerly known as *U2af1-rs1*) in mESCs, an effect linked to increased cellular passage in conventional media [53]. Imprinting defects on mESCs were then found not to be restricted to this locus but were also seen in *Igf2r/Airn*, *Igf2-H19* and *Mest* loci with a significant degree of heterogeneity between mESC lines. Importantly, these imprinting defects persisted in mESC-derived animals or cloned mice generated by nuclear transfer of imprinting-defective mESCs being associated with aberrant developmental phenotypes [54,55]. These findings suggest that imprinting stability at specific loci in mESCs may vary upon both derivation and subsequent propagation of mESC lines in conventional media. Later on, biological sex was also found to drastically affect imprinting fidelity. Zvetkova et al. [56] reported for the first time that hypomethylation at imprinted regions was commonly seen in female but not in male mESCs [56]. This seems to be a consequence of the fact that female mESCs exhibit lower global methylation levels compared with male ESCs [12,56]. It is now well known that low DNA methylation levels in female mESCs are explained by the presence of two active X chromosomes (XaXa), which is linked to the overdosage of the X-linked *Dusp9* gene. *Dusp9* overexpression induces a reduction in MAPK/ERK signalling, which in turn down-regulates DNMT activity, leading to global reduction of DNA methylation levels from which imprints are not spared [57,58]. Besides the impact of biological sex, more recently, a study identified a genetic determinant linked to gain of methylation at the *Dlk1-Dio3* cluster in developmentally compromised male mESCs [59], suggesting that epigenetic states at imprinted genes can be modulated by genetic variants. Importantly, although imprints could sometimes fail in conventional mESCs, this model proved to be meaningful for the understanding of imprinting regulation at specific loci, such as *Igf2/Airn* and *Dlk1-Dio3* clusters [60,61].

In 2007, scientists isolated mouse epiblast stem cells (mEpiSCs) from the post-implantation blastocysts, which are in a primed pluripotency state [47]. These cells rely on FGF2 and Activin A for *in vitro* culture, which differ from the LIF-based requirements of mESCs. Epigenetically, these cells have high global DNA methylation levels, and, in female cells, X-chromosome inactivation (XCI) has been established, and therefore, they are XaXi. Interestingly, the hypomethylation trend commonly seen for XaXa mESCs was not seen in female mEpiSCs [62]. In fact, these more developmentally advanced PSCs have been reported to exhibit more stable imprints than conventional mESCs [62,63]. However, they have not been as extensively screened as mESCs. If confirmed, it remains unclear whether this stability arises from reduced epigenetic turnover linked to their post-implantation origin or specific media formulations.

A year after the isolation of mEpiSCs, Smith and colleagues made a groundbreaking discovery of a media formulation that enables the derivation and maintenance of mESCs in a homogeneous ground state of pluripotency, commonly referred to as the naive state [45]. Indeed, naive mESCs are able to generate healthy chimeric mice and even all-mESC mice by tetraploid complementation more efficiently than conventional conditions [64]. However, these properties are lost upon prolonged FGF/ERK inhibition by Mek1/2 suppression, presumably due to global DNA hypomethylation and loss of DNA methylation at imprinted loci [65,66]. Safeguarding imprints under the low DNA methylation levels of naive pluripotency is essential to enable mESCs in a ground state of pluripotency to fully capture their developmental potential.

In summary, the sustainability of the different pluripotent states *in vitro* renders imprints susceptible to errors, which are major in naive conditions, relatively minor in conventional conditions and mostly stable in primed pluripotency conditions (Figure 2B).

### Imprinting status in mouse iPSCs

Despite the impact of imprinting defects on the developmental potential of mESCs, they remained largely overlooked in stem cell research. Interest reemerged with the advent of iPSCs and the initial discoveries of reprogramming-specific imprinting defects. The first imprinting defect specifically linked to mouse iPSCs (miPSCs) generated under conventional conditions was the aberrant acquisition of DNA methylation at the maternal ICR of the *Dlk1-Dio3* cluster [67,68]. This epigenetic abnormality resulted in the silencing of several maternally expressed non-coding RNAs significantly compromising the developmental potential of these cells, impairing their ability to contribute to chimaeras and to generate animals derived entirely from iPSCs [67]. This epigenetic abnormality was therefore signalled as a biomarker of partially reprogrammed iPSCs [68]. The first explanation for the origin of this defect blamed the stoichiometry of the reprogramming factors used. Carey et al. [69] showed that when reprogramming factors were provided in a different stoichiometric ratio (higher OCT4/KLF4 and reduced c-MYC/SOX2), the resulting iPSCs retained proper genomic imprinting at the *Dlk1-Dio3* region, which correlated with better developmental outcomes [69]. Later on, Stadtfeld et al. [70] challenged this view by showing that this stoichiometry is not totally inert to errors on the *Dlk1-Dio3* region. Instead, the authors provided a correction mechanism for these hypermethylation defects based on the addition of ascorbic acid (AA), also known as vitamin C, to LIF/serum media [70]. They proposed that AA worked as a cofactor of TET enzymes, increasing their enzymatic activity which was fundamental to maintain the unmethylated status of the maternal ICR at the *Dlk1-Dio3* region.

Subsequent research extended the repertoire of imprinting defects to other imprinted loci in conventional miPSCs [12,62,63,71]. Importantly, these defects affected imprinted regions differently and were shown to vary among different iPSC lines, highlighting overall instability of imprinting during iPSC reprogramming. Yet, the different studies consistently showed the same consensus trend: while paternally inherited imprints such as *Dlk1-Dio3* and *Igf2-H19* are highly susceptible to regain methylation at the unmethylated allele, maternally inherited imprints remain mostly stable. Such behaviour suggests that the mechanisms governing imprinting stability during reprogramming may differ between paternally and maternally inherited imprints.

**Figure 3:**
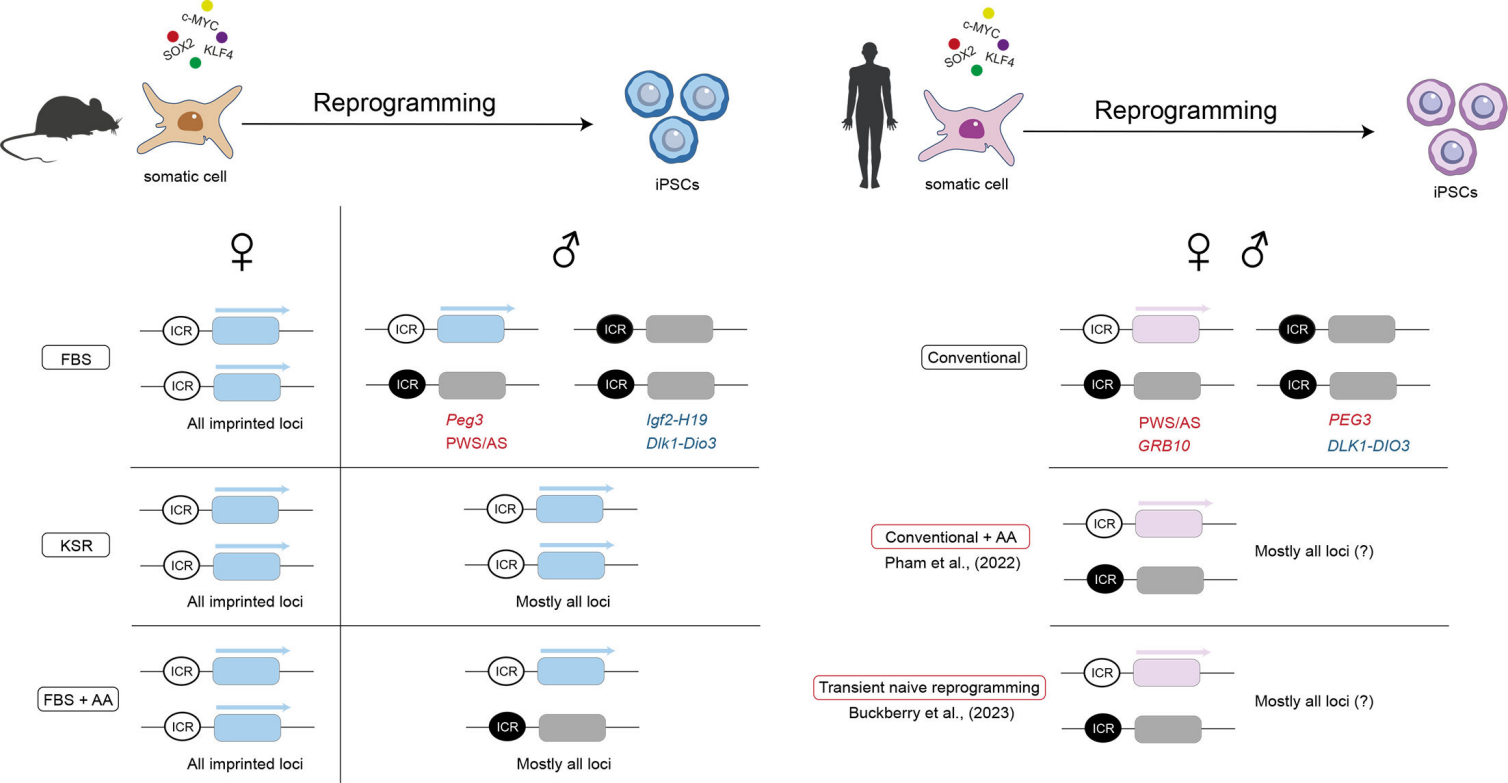
Imprinting status in mouse and human iPSCs following reprogramming. Schematic representation of imprinting status of female and male induced pluripotent stem cells (iPSCs) upon reprogramming of both mouse and human somatic cells. The left panel depicts the imprinting outcomes in female and male mouse iPSCs generated under different culture conditions (FBS, KSR, and FBS + AA). The right panel illustrates the imprinting outcomes in female and male human iPSCs derived under conventional conditions, conventional + addition of AA [74] and transient naive reprogramming [75]. In blue are represented examples of paternal imprinted loci and in red examples of maternal imprinted loci that typically show the imprinting defects depicted (please refer to the main text for other examples); black circles: methylated ICR; white circles: unmethylated ICR. AA - ascorbic acid; FBS, foetal bovine serum; ICR - imprinting control region; KSR, knockout serum replacement; PWS/AS - Prader–Willi Syndrome/ Angelman Syndrome imprinted locus.

In the initial studies, little attention was made towards the biological sex of the cells studied. Sun et al. [62] provided the first study on the impact of the biological sex of cells on imprinting stability of miPSCs. They showed that, similarly to female mESCs, female miPSCs present lower methylation levels at most imprinted regions than male counterparts [62]. Again, this hypomethylation was linked to the XaXa status of female miPSCs [72,73]. In our recent systematic study, we confirmed that female miPSCs, which reactivated the inactive X during reprogramming, consistently lost imprints at all regions studied and this resulted in widespread loss of monoallelic expression of imprinted genes [12] (Figure 3). These findings confirm a role for X-chromosome reactivation in driving imprinting instability in female cells.

Media formulation is another variable affecting imprinting stability during reprogramming. In fact, we observed contrasting imprinting defects in male miPSCs generated under standard FBS or chemically defined knockout serum replacement (KSR) conventional media. While FBS-derived iPSCs showed correct imprints on most imprinted regions, with the notable exception of hypermethylation defects at paternally imprinted *Dlk1-Dio3* and *Igf2-H19*, KSR-derived iPSCs showed a reduction of methylation at various imprinted regions [12]. These contrasting effects of FBS and KSR media led us to attempt new media formulation strategies to try to mitigate imprinting defects. While a 1:1 mix of KSR/FBS successfully amended the hypermethylation at the *Igf2-H19* cluster and, to a lesser extent, at the *Dlk1-Dio3* cluster, it induced loss of methylation in the maternal imprints. In contrast, the addition of AA, a component of the KSR formulation, to the FBS media slightly improved the hypermethylation defects of both *Igf2-H19* and *Dlk1-Dio3,* while not affecting the maternal imprints [12]. Taken together, these data undoubtedly suggest that the addition of AA to conventional serum-containing media formulation is advisable to limit the propagation of hypermethylation defects at the paternally imprinted regions, not only in miPSCs but also in mESCs [12,59,61,70].

As mentioned above, the stoichiometry of reprogramming factors is a variable to take into consideration to prevent imprinting errors from accumulating during reprogramming [69]. Notably, a study demonstrated that excluding OCT4 from the reprogramming cocktail, although decreasing the efficiency of iPSCs generation, had a positive effect on imprinting maintenance [76]. Interestingly, reprogramming factors, such as OCT4 and SOX2, have binding sites at affected imprinted regions such as the *Igf2-H19* cluster [77], suggesting their potential to influence local epigenetic profiles. Although further research is needed, maintaining intact imprints during miPSC reprogramming may depend on the combination of the right media composition, an appropriate AA concentration, and the correct dosage of reprogramming factors.

### Imprinting status in human ESCs

Early studies demonstrated that hESCs are largely able to preserve genomic imprinting during *in vitro* culture [14,78–83]. Instead of being equivalent to conventional mESCs, hESCs more closely resemble mEpiSCs. Both hESCs and mEpiSCs rely on similar signaling pathways in culture, have comparable epigenetic landscapes, including the XaXi status in female cells, which are typical of the primed pluripotency state (Figure 2A). Since female hESCs have undergone XCI, they do not show meaningful differences in global DNA methylation levels compared with male hESCs and, to date, no sex differences in imprinting stability have been documented. Despite the substantial degree of imprinting stability, some hESCs did show loss of allele-specific expression of particular genes, such as *H19, IGF2* and *MEG3* [78,81,83]. In one big cohort study, the authors found a variation of *IGF2* allelic expression in the same hESC line cultured at different laboratories [83]. A possible explanation for this variability is the prolonged culture where double expression of *IGF2*, encoding for insulin-like growth factor 2, could confer a selective advantage in culture. This specific case exemplified that subtle variations become more evident when analysing a larger set of cell lines, emphasising the heterogeneity inherent to these lines [83].

These initial studies were mostly based on transcription analysis, while methylation analysis, when performed, was scarce and locus-specific. Subsequent studies such as the one by Nazor et al. [13] approached the issue of imprinting stability in primed hESCs using DNA methylation arrays. Surprisingly, their results showed that imprinted loci exhibited frequent DNA methylation aberrations, with recurrent hypermethylation found at genes such as *DIRAS3*, *NAP1L5*, *MEST*, *H19*, and *PEG3*, and occasional hypomethylation at *PLAGL1* and *GRB10*. These imprinting abnormalities were found to affect gene expression, notably silencing *H19* and *PEG3* genes [13]. By using IMPLICON, an amplicon-sequencing method designed to measure DNA methylation specifically at multiple ICRs, we corroborated these results, notably at the *IGF2-H19* and *PEG3* imprinted clusters [84]. Importantly, Nazor et al. [13] showed that imprinting defects at specific loci persist during hESC differentiation, raising some concerns regarding the safety and reliability of hPSCs for their downstream applications. However, the extent to which these defects affect functionality remains to be fully understood and may depend on the specific context.

Like for mESCs, hESCs were also reset into the naive pluripotent state [46,85,86]. This achievement marked a significant breakthrough as it provided a new platform to study early human developmental processes [87]. However, as with mESCs, hESCs in the naive state also exhibit generalised loss of imprints *in vitro* [84,88] (Figure 2B). While this loss of imprinting represents a deviation from their *in vivo* counterparts, the extent to which imprint instability affects developmental modelling and clinical utility remains to be fully understood and likely depends on the specific imprints affected and applications involved. Efforts to correct imprinting defects in naive hESCs have been recently explored by Fischer et al. [89]. In this study, the authors showed that either reducing the MEK/ERK inhibition or overexpressing the ZFP57 KRAB zinc-finger protein, a known imprint-protecting factor, during primed-to-naive resetting could partially preserve imprinting at a subset of loci, while generating *bona fide* naive hESCs [89]. Importantly, the combination of these two approaches showed to confer greater imprint protection than either strategy alone. While this represents a significant advance in improving the imprinting fidelity of naive hPSCs, this combined approach has so far been validated at only the PWS/AS imprinted locus [89]. Further refinement of naive induction protocols is necessary to ensure the preservation of imprint integrity across the full repertoire of loci.

### Imprinting status in human iPSCs

With the studies of Stadtfeld et al. [67] on reprogramming-induced imprinting defects in miPSCs, researchers quickly turned to investigate whether the same issue applied to human iPSCs (hiPSCs) [13,84,90,91]. Not surprisingly, the first systematic studies using array-based DNA methylation analysis reported recurrent imprinting defects in hiPSCs [13,90,91]. In general, their findings support the idea that hiPSCs are more prone to imprinting errors than hESCs, a conclusion later confirmed by a systematic study using allele-specific RNA-seq data [14]. All these studies also demonstrated that some genes are more prone to imprinting errors than others. This tendency was linked to the parent-of-origin methylation regulating their monoallelic expression. The overall picture suggested that paternal imprints such as *IGF2-H19* and *DLK1-DIO3* were particularly prone to be more affected in hiPSCs by hypermethylation, while maternal imprints were mostly stable with a rarer tendency for losing methylation (Figure 3). A notable exception was the maternally methylated *PEG3* locus which was found to be persistently hypermethylated [13,84,91]. Importantly, these defects were not limited to hiPSCs, but also found in some hESCs, suggesting that, besides reprogramming, culture conditions may also play a role in inducing imprinting aberrations. This complicates the distinction between the effects of reprogramming and those of prolonged culture on imprinting errors. Nazor et al. [13] have looked at low- and high-passage iPSCs. They documented that errors in *DLK1-DIO3* and *PEG3* clusters were already present in low-passage cells, whereas errors at the *IGF2-H19* locus were only found at higher passages [13]. They proposed that errors in low-passage cells were reprogramming-induced, while those in high-passage cells arise from culture conditions. However, the precise dynamics of imprinting dysregulation during hiPSC reprogramming remain to be systematically assessed.

Efforts to correct imprinting defects in hiPSCs were recently explored by Pham et al. [74], whose focus was establishing hiPSCs as suitable cellular models for imprinting diseases like Silver-Russell syndrome [74]. Inspired by the Stadtfeld et al. [70] approach in miPSCs, the authors supplemented the culture medium with AA to correct hypermethylation at the *IGF2-H19* and *DLK1-DIO3* imprinted regions (Figure 3). Importantly, this approach was only feasible when hypoxia was used, since normoxia triggered spontaneous differentiation. These findings highlight the potential for culture conditions to affect imprinting fidelity in hPSCs. However, the extent of imprinting correction remains to be further explored since only 7 imprinted regions were assessed and the long-term effects of AA addition on hiPSC pluripotency have not been investigated.

With the emergence of the naive formulation for hESCs, researchers started reprogramming somatic cells under naive conditions [92–94] to generate hiPSCs in the ground state of pluripotency with no signs of somatic epigenetic memory. As predicted, naive hiPSCs become globally hypomethylated and exhibit loss of imprinting [92]. Interestingly, loss of imprinting seems to be a rather late event in the reprogramming process [75]. Considering this, Buckberry et al. [75] developed a transient naive reprogramming strategy to generate hiPSCs that effectively erase the somatic cell epigenetic programme while preserving intact imprints [75]. This strategy begins with a naive formulation at the first stages of reprogramming, followed by a shift to a primed formulation at later stages. While this approach opens new avenues towards improved hiPSC models, its dependence on the current primed media formulations does not ensure to fully resolve imprinting defects in hPSCs.

## Concluding remarks

As summarised here, imprinting defects in both mouse and human PSCs remain unstable and sensitive to current reprogramming and media formulations. While this may not be exclusive to PSCs, higher methylcytosine turnover seen in PSCs compared with somatic cells [95] may destabilise imprinting in this dynamic environment associated with both naive and primed pluripotency states, especially upon extended *in vitro* culture. These findings underscore the challenges of maintaining a controlled environment in which PSCs retain proper imprinting while preserving their full developmental potential.

Once imprinting defects arise in PSCs, they persist throughout their maintenance and differentiation, being present in their derived differentiated products [12,13,96]. A paradigmatic example is the presence of imprinting defects in cell products used in the first iPSC-based transplantation trial [96]. Although the significance of these defects for iPSC applications is still poorly understood, they raise valid concerns about the safety and reliability of certain iPSC-derived products, underscoring the need for careful evaluation on a case-by-case basis. One obvious concern lies in modelling imprinting disorders, where imprinting changes can reshape the original epigenetic and transcriptional landscape of a specific disease or even convert healthy hPSCs into an imprinting disease-like state [97]. Notably, for non-imprinting diseases, imprinting defects can still compromise the reliability of hPSC models, as imprinted gene dosage influences cellular fitness and differentiation in specific subtypes—for instance, *IGF2* in haemopoietic commitment [15] and *MEG3* in neuronal differentiation [16]. These examples highlight the potential for imprinting defects to undermine the reliability of stem cell-based organoid models, which are increasingly employed in disease modelling and drug discovery [98]. This concern is amplified in cell therapies, where imprinting errors may disrupt normal gene regulation, potentially leading to aberrant functionality, developmental abnormalities, increased tumourigenic risk, or other unintended consequences in transplanted tissues. Importantly, the effects of imprinting defects are likely to be cell- or tissue-specific, highlighting the need for further research to evaluate their associated risks across different cell types or tissues.

For stem cell epigeneticists, the key question remains: how can imprinting errors be prevented or mitigated during stem cell reprogramming and culture in naive and/or primed pluripotency states? Despite the efforts that have been made throughout the years, correcting imprinting defects in PSCs remains a challenge. In primed cells, imprinting errors often involve hypermethylation in specific imprinted regions, whereas in naive cells, methylation is largely lost across most regions. For primed PSCs, modulation of TET activity has been identified as a mitigation strategy to counteract hypermethylation in imprinted regions [70,74]. In contrast, in naive PSCs, modulation of the MEK/ERK pathway combined with overexpression of the imprint-protecting factor ZFP57 has recently emerged as a promising strategy to, at least, mitigate imprinting erasure in these cells [89]. In both cases, culture conditions require further optimisation to support stable imprint maintenance.

Developing robust methods to safeguard imprinting during reprogramming and culture of PSCs is crucial for advancing the stem cell field. Protecting imprinting fidelity ensures the epigenetic integrity of PSCs and enhances their reliability for both basic research and therapeutic applications. Greater awareness and concerted efforts within the scientific community to understand, prevent and mitigate these defects will be instrumental in unlocking the full potential of PSCs. This progress will pave the way for their broader use in disease modelling, drug discovery and regenerative medicine, ultimately improving the impact and safety of stem cell-based innovations.

**Perspectives**

- Pluripotent stem cells (PSCs) can self-renew and differentiate into almost any cell type, making them invaluable for research and therapy. However, they are prone to errors in genomic imprinting, which governs the parent-specific expression of certain genes. These imprinting errors persist through differentiation, possibly undermining the consistency of PSC-derived cells for disease modelling and regenerative medicine. Ensuring genomic imprinting integrity during PSC reprogramming and culture is therefore essential to maintain their reliability in downstream applications.
- Imprinting defects in mouse and human PSCs are unstable and influenced by reprogramming methods and media formulations. Different pluripotency states expose cells to varying epigenetic landscapes which affect imprinting: primed cells often show hypermethylation in specific imprinted regions, while naive cells exhibit widespread loss of methylation globally and at imprinted regions. These challenges highlight the difficulty of maintaining a controlled environment where PSCs preserve proper imprinting and full developmental potential.
- Preventing or mitigating imprinting errors during PSC reprogramming and culture, in both naïve and primed states, remains a significant challenge with no definitive solution. Understanding the mechanisms behind these errors is vital to develop methods that safeguard imprinting and preserve PSC epigenetic integrity. This will enhance the reliability of PSCs for research and therapeutic applications, improving the safety and impact of stem cell-based innovations. Advancements in this area will drive progress in regenerative medicine and stem cell biology.

## Abbreviations

AA: Ascorbic Acid
AS: Angelman Syndrome
DMRs: Differentially Methylated Regions
DNMT: DNA methyltransferase
ESCs: embryonic stem cells
FBS: Foetal Bovine Serum
ICM: Inner cell mass
ICR: imprinting control region
KSR: Knockout Serum Replacement
LIF: Leukemia Inhibitory Factor
PSCs: pluripotent stem cells
PWS: Prader-Willi Syndrome
TET: ten-eleven translocation
XCI: X-chromosome Inactivation
iPSCs: induced pluripotent stem cells
sDMRs: Somatic differentially methylated regions
